# Post-fire seed dispersal of a wind-dispersed shrub declined with distance to seed source, yet had high levels of unexplained variation

**DOI:** 10.1093/aobpla/plac045

**Published:** 2022-10-06

**Authors:** Cara Applestein, T Trevor Caughlin, Matthew J Germino

**Affiliations:** Department of Biology, Boise State University, 1910 W University Drive, Boise, ID 83725, USA; Department of Biology, Boise State University, 1910 W University Drive, Boise, ID 83725, USA; US Geological Survey, Forest and Rangeland Ecosystem Science Center, 230 N Collins Rd, Boise, ID 83702, USA

**Keywords:** Data fusion, ecological forecasting, hierarchical Bayesian models, natural regeneration, seed dispersal

## Abstract

Plant-population recovery across large disturbance areas is often seed-limited. An understanding of seed dispersal patterns is fundamental for determining natural-regeneration potential. However, forecasting seed dispersal rates across heterogeneous landscapes remains a challenge. Our objectives were to determine (i) the landscape patterning of post-disturbance seed dispersal, and underlying sources of variation and the scale at which they operate, and (ii) how the natural seed dispersal patterns relate to a seed augmentation strategy. Vertical seed trapping experiments were replicated across 2 years and five burned and/or managed landscapes in sagebrush steppe. Multi-scale sampling and hierarchical Bayesian models were used to determine the scale of spatial variation in seed dispersal. We then integrated an empirical and mechanistic dispersal kernel for wind-dispersed species to project rates of seed dispersal and compared natural seed arrival to typical post-fire aerial seeding rates. Seeds were captured across the range of tested dispersal distances, up to a maximum distance of 26 m from seed-source plants, although dispersal to the furthest traps was variable. Seed dispersal was better explained by transect heterogeneity than by patch or site heterogeneity (transects were nested within patch within site). The number of seeds captured varied from a modelled mean of ~13 m^−2^ adjacent to patches of seed-producing plants, to nearly none at 10 m from patches, standardized over a 49-day period. Maximum seed dispersal distances on average were estimated to be 16 m according to a novel modelling approach using a ‘latent’ variable for dispersal distance based on seed trapping heights. Surprisingly, statistical representation of wind did not improve model fit and seed rain was not related to the large variation in total available seed of adjacent patches. The models predicted severe seed limitations were likely on typical burned areas, especially compared to the mean 95–250 seeds per m^2^ that previous literature suggested were required to generate sagebrush recovery. More broadly, our Bayesian data fusion approach could be applied to other cases that require quantitative estimates of long-distance seed dispersal across heterogeneous landscapes.

## Teaser

Sagebrush seeds are dispersed naturally from plants that survive wildfires and can also be seeded by natural resource agencies to aid restoration. There is a need to better understand natural dispersal patterns to avoid spending limited resources for restoration on areas with adequate natural seed dispersal. Researchers conducted a seed trapping study around sagebrush patches post-fire and used a novel modelling approach to estimate dispersal distances. They found that most seed travels less than a few metres and hardly ever farther than 16 m and that seed density from natural dispersal may often be inadequate for restoration.

## Introduction

Seed dispersal sets the spatial template for patterns of plant-population recovery across disturbed landscapes (Russell and Roy 2008; Caughlin et al. 2016; Snell et al. 2019; Gill et al. 2020). Seedling recruitment after disturbance is often related to proximity to seed sources (Webber et al. 2010; Leirfallom et al. 2015). Seed-source patches in disturbed areas drive recolonization, including expansion of remnant islands as new recruits establish around existing reproductive plants (Corbin and Holl 2012). To predict how and where plant populations will re-establish after disturbance, we need to understand the sources of heterogeneity in seed dispersal events (Clark et al. 1999; Ozinga et al. 2005; Caughlin et al. 2014; San-José et al. 2019).

Small-scale spatial heterogeneity in post-disturbance seed dispersal can be a major determinant of plant population recovery (DiVittorio et al. 2007). Understanding this heterogeneity through spatially explicit seed dispersal predictions can inform spatial prioritization of limited restoration resources and thus cost-effectiveness of restoration (Neeson et al. 2015; Jones et al. 2018; Strassburg et al. 2020). Many past seed trapping experiments needed to make these sorts of dispersal predictions have focused on intensive trapping at short distances from seed sources (Greene and Calogeropoulos 2002). Longer distance travel of seeds across landscapes is rare and difficult to detect via experimental methods; however, it is hypothesized to have an oversized impact on plant colonization (Clark et al. 1998; Cain et al. 2002). For instance, a prior study on dispersal of an invasive plant using seed traps found that mean dispersal distance was only 0.26 m, an insufficient distance to explain the continental scale of ongoing range expansion; models demonstrated that only one-in-a-million seeds moving kilometres further than the mean was sufficient to replicate the observed distribution of the plant (Neubert and Caswell 2000). These infrequent, but critically important, long-distance dispersal events challenge field-based methods for quantifying dispersal distance.

Previous researchers have modelled how seed density decreases with distance from remnant seed sources in many disturbed landscapes, including heathlands, tropical forests and subalpine forests (Hammill et al. 1998; Holl 1999; Gill et al. 2020). These models can help answer questions about whether or not seeds will arrive at certain landscape locations and where to prioritize direct seeding for restoration (Peeler and Smithwick 2020). However, variability in seed dispersal during succession contributes to model uncertainty (e.g. Shive et al. 2018) and disentangling the sources of variability will be necessary to operationalize models for restoration decision support.

Direct seeding (‘active restoration’) of desired species is common practice on disturbed landscapes to increase the pace of natural regeneration and ensure that propagules of desired species arrive before or at least concurrently with invasive species (Palma and Laurance 2015). However, when disturbed landscapes are not seed-limited, supplemental seedings can be ineffective at increasing the rate of vegetative recovery or even suppress natural regeneration (Schoennagel and Waller 1999; James and Svejcar 2010; Peppin et al. 2010).

Wind is a common agent of seed dispersal across many different ecosystems and taxa (Nathan et al. 2011; Sullivan et al. 2018). Wind strength and direction vary seasonally and the timing of major wind events in relationship to the timing of seed ripening can have significant effects on dispersal distances (Heydel et al. 2015). Furthermore, seed functional traits, landscape characteristics and weather can all affect wind-driven dispersal of seed across landscapes. Seeds with specific wind dispersal mechanisms, such as a pappus or wings, have a higher propensity towards long-distance or widespread seed dispersal (Ozinga et al. 2005; Dauer et al. 2007; Tamme et al. 2014). Small seed mass can also contribute to longer wind dispersal distances (Hoppes 1988; Tamme et al. 2014). Additionally, wind energy for seed dispersal can be both constrained and/or modified by landscape characteristics including canopy density and structure (Nathan et al. 2009), which can be particularly heterogeneous in disturbed areas.

Sagebrush steppe provides an excellent system for studying how wind-driven seed dispersal from remnant patches varies across scales because these ecosystems are experiencing unprecedented habitat disruption from megafires (Miller et al. 2011) and tens of millions of dollars are spent each year on burned area rehabilitation, particularly purchasing of sagebrush seed (as a representative example, the US Bureau of Land Management allocated $20 million USD to burned area rehabilitation in Fiscal Year 2018). Sagebrush is considered a keystone species in these ecosystems, as the shrub supports subsequent recovery of many wildlife and plant species (Beck et al. 2012). Investment in aerial seeding of sagebrush assumes that sagebrush regeneration is primarily limited by seed availability owing to short longevity of the sagebrush seed bank (Wijayratne and Pyke 2012). The capacity for unburned remnants or edges to provide seed is relatively unknown and implicitly assumed to be negligible. While several studies have examined post-fire regeneration of big sagebrush, these studies have not specifically addressed the impact of unburned remnant patches (or newly created patches) within a larger burn context (DiCristina and Germino 2006; Lesica et al. 2007; Ziegenhagen and Miller 2009; Nelson et al. 2014). Young and Evans (1989) and Welch and Nelson (1995) asserted that seed dispersal distances of sagebrush stands are <1–2 m from the maternal plant (Young and Evans 1989; Welch and Nelson 1995). Despite this, seedling recruitment can occur several hundred metres from remnant adults into burned areas and on unseeded landscapes (Mueggler 1956; Nelson et al. 2014).

Our questions in this study were:

(1) How far do sagebrush seeds disperse and how variable is sagebrush seed dispersal?
(2) Which landscape scales best explain variation in seed dispersal (trap, transect, patch, site)? Do wind-direction metrics help explain variation in seed dispersal?
(3) How does seed dispersal from seed-source patches compare with aerial seeding rates?

## Methods

We conducted a seed trapping study around sagebrush patches during the winters of 2018/19 and 2019/20. Our vertical wind traps were designed to catch seeds at any height in the wind from the ground to approximately the height of release (i.e. the height of flowers on seed-source plants). Big sagebrush flowers in the fall (typically November, depending on the elevation and weather) and seeds mature and release in early to mid-winter. Seeds weigh 0.25 mg or less and are approximately 1.5 mm in diameter (Jacobs et al. 2011). Seed traps were arrayed on two transects per patch of sagebrush plants that were adjacent to (or surrounded by) areas with no sagebrush and instead were dominated by grasses. Multiple patches (and thus, transects) were evaluated in each of six sites. Three of the sites were sampled in the first year of the study and the three other sites were sampled in the second year. We evaluated seed dispersal under and away from sagebrush patches.

We used vertical seed traps as opposed to ground traps for several reasons. First, sagebrush seed dispersal occurs during the winter when snow cover may be present. Our small ground traps directly beneath the canopy were fairly sheltered from snow but any ground traps set outside of the canopy would have accumulated snow and been non-functional. Secondly, we anticipated that seed density would be very low and that we would therefore need a large trap area to capture seeds. Creating greater surface area for vertical traps was more feasible than for ground traps. We account for our trap design using a novel modelling approach with a latent variable for ground distance term (see below).

### Sites

**Figure 1.**
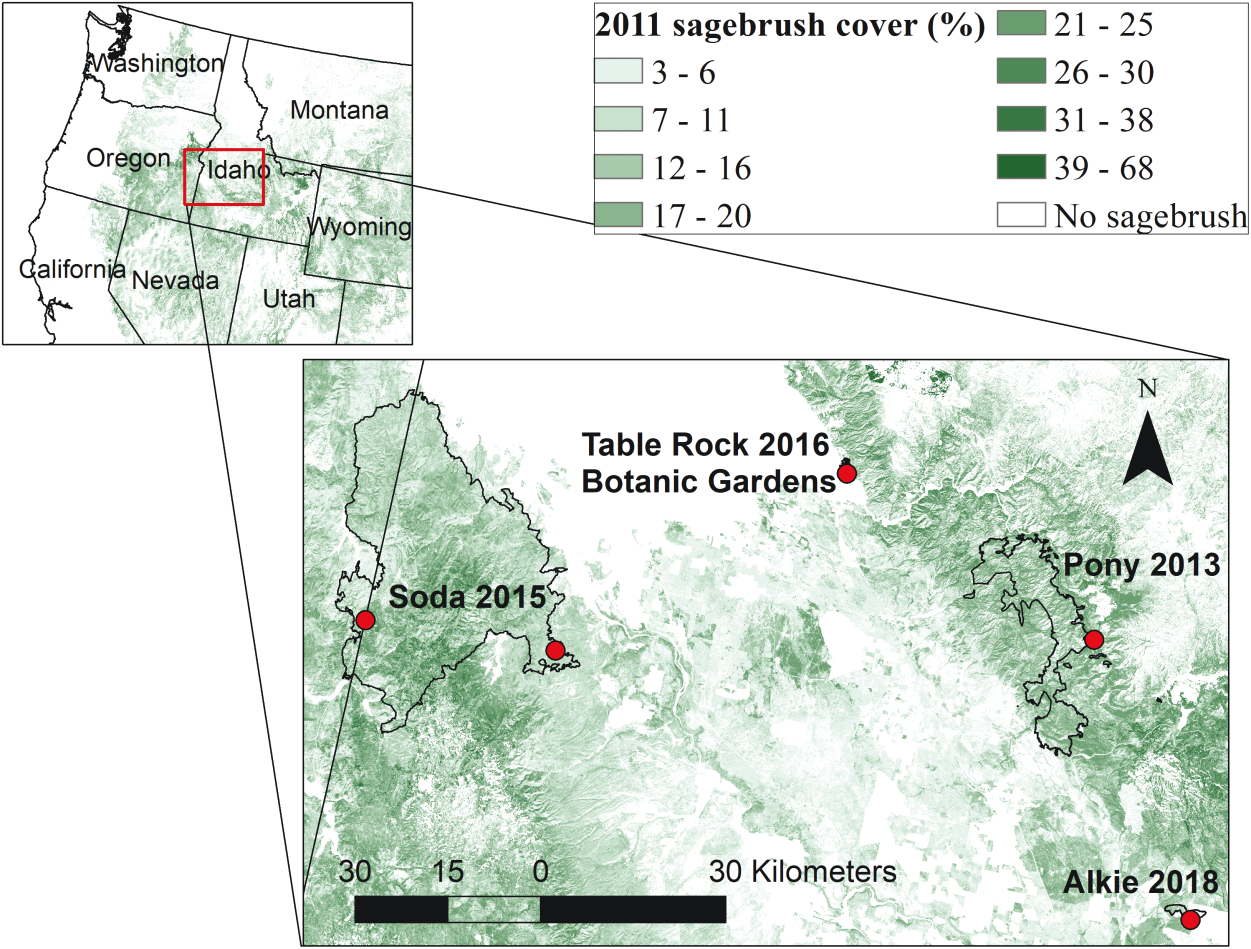
Locations of fires (outlines) and trapping sites (dots) for dispersal study shown as an inset map on western USA and 2011 sagebrush cover (%) from the National Land Cover Database (NLCD) (Rigge et al. 2020).

Study sites for the first year of trapping were the Soda Wildfire (113 kha, burned 2015), Alkie Wildfire (814 ha burned 2018) and the Botanical Garden in Boise (at a planted sagebrush patch in a disturbed area otherwise dominated by grasses). Study sites for the second year of trapping were the Soda Wildfire, the Pony Wildfire (60 kha, burned 2013) and Table Rock fire (1 kha, burned 2016) (Fig. 1). The two trapping locations on the Soda Wildfire were at different locations on the fire (Year 1 location in the southeast, Year 2 location in the central west) and thus were considered separate sites. The seed trap size, dates of trapping and site summary information, including sample sizes, are given in Table 1.

**Table 1.** Number and sizes of seed-collection traps, their spatial deployment and trapping dates by year. Alkie was excluded due to seed crop failure and no seeds trapped. The total number of vertical patches and traps excludes those lost to animals or weather.

	Year 1	Year 2
Vertical trap size (cm)	50 × 91 rectangle	50 × 76 rectangle
Under-crown trap size (cm)	25.4-cm radius pan (with 2.5-cm centre hole)	10 × 10 square
Trap distances (m)	2, 4, 7, 10, 13	2, 4, 6, 10, 14, 18, 22, 26
Sites	Soda, Botanical Garden, Alkie (excluded)	Soda, Table Rock, Pony
Number of patches	4	15
Total number of vertical traps	36	237
Total number of under-crown traps	6	30
Dates of collection		
Soda	24 November 2018 to 21 December 2018	Round 1: 22 November 2019 to 17 December 2019; Round 2: 17 December 2019 to 10 January 2020
Botanical Garden	4 December 2018 to 22 December 2018	–
Alkie	26 November 2018 to 3 January 2019	–
Table Rock	–	Round 1: 22 November 2019 to 14 December 2019; Round 2: 14 December 2019 to 6 January 2020
Pony	–	Round 1: 23 November 2019 to 18 December 2019; Round 2: 18 December 2019 to 7 January 2020

Patches (*n* = 22) were selected by reconnaissance at each site based on the following criteria: there had to be at least five individual reproductive plants in each patch, slopes in and around the patches had to be less than 20° and patches had to be isolated enough from other patches so that no other seed-bearing sagebrush plants in the surrounding area could be any closer to the traps than the individuals in the patch. In a few cases, all flower stalks were clipped from single individual sagebrush that were located outside of a patch to satisfy these criteria. Patches could either be unburned remnants or created from planting seedlings or aerial seeding.

Most sites were dominated by *Artemisia tridentata* ssp. *wyomingensis*, although the dominant subspecies at the Pony wildfire site was *A. tridentata* ssp. *xericensis.* The surrounding vegetation for the sites during the first year was exotic annual grasses at the Soda site, a mixture of perennial and annual grasses at the Botanical Garden, and the Alkie site was freshly burned and had no vegetative cover. The surrounding vegetation for sites during the second year was mixed low sagebrush (*Artemisia arbuscula*) and low-statured grasses at the second Soda fire site, exotic annual grasses at Table Rock and mixed low sagebrush and low-statured grasses at the Pony site.

### Seed traps

During the first winter, traps (*n* = 79) were located under canopy, 2 m, 4 m, 7 m, 10 m and 13 m from the patch. Since seeds were found at all distances in the first year, we increased the distance of the farthest traps in the second year. During the second winter, traps (*n* = 275) were located under canopy, 2 m, 4 m, 6 m, 10 m, 14 m, 18 m, 22 m and 26 m from the patch. Traps were arranged along two transects per patch (except for the one patch at the Botanical Gardens, for which there were four transects) with angles chosen based on the following criteria: first, all transects had to be isolated enough so that no reproductive individuals were any closer to the traps than the plants in the patch. Given this requirement, the first angle was aligned as close as possible against the prevailing wind direction at the site and the second angle was aligned as close as possible towards the prevailing wind direction at the site (these wind directions were taken from prior year weather station data—actual wind directions during trapping season were not always as expected). Trap distances were measured from the base of the individual reproductive individual sagebrush plant where each transect began (termed ‘base individual plant’ below).

Vertical traps were constructed from two 5 × 5 cm wooden stakes that were either 1.23 m tall (Year 1) or 0.91 m tall (Year 2). The stakes were set 50 cm apart with 0.55 oz white AgFabric (Wellco Industries, Corona, CA, USA) stapled between the stakes **[see****Supporting Information—Fig. 1****]**. The AgFrabic was then sprayed with Tanglefoot (Scotts Miracle Gro, Grand Rapids, MI, USA) to provide a persistently adhesive surface. Under-crown traps were circular cake Bundt pans (25.4-cm radius with 2.5-cm centre hole) filled with marbles to prevent seeds from blowing out (Year 1) or square 10 × 10 cm frames with sprayed AgFabric stapled on (Year 2) and were set directly under the crown of the base individual plant. Some vertical traps failed because of weather or animal interference (including all traps at three of the six patches at Pony) and these were excluded from analysis, resulting in some missing data values. Excluding Alkie and failed traps, the total sample size was 5 sites, 19 patches, 40 transects and 309 traps.

### Patch characteristics

At each patch, we recorded the following information for 10 individual plants (or all plants if the patch was composed of fewer than 10 plants): number of flowering stalks, and average length of flowering stalks (of three representative stalks). If there were more than 10 individual plants in the patch, the first two plants measured were the base individual plants for the transects, then the three tallest plants in the patch, then five additional representative plants. If there were fewer than 50 plants in a patch, the number of reproductive and non-reproductive plants was counted directly. If there were more than 50 plants in a patch, we estimated number of individuals by counting the number of plants in randomly distributed subplots (the number of which were proportional to the size of the patch) and scaling this number up to the patch size. We also visually estimated surrounding vegetation canopy height in bins outside of the patch (<30 cm, 30–50 cm, 50–75 cm, >75 cm), which was used to parameterize the WALD wind model.

### Estimating maximum seed production

We estimated maximum seed production per individual by multiplying number of stems by the average stem length by 8.2 (mean number of flower heads per 1-cm stalk length) by 3.7 (mean flowers per head). The mean number of flower heads and mean flowers per head were taken from Winward and Tisdale (1977) morphological measurements on *A. tridentata wyomingensis.* Seed production was estimated during the same season as trapping (upon trap deployment).

### Terminal velocity

We collected samples of sagebrush seeds from reproductive plants at each site in areas outside of the patches for assessment of terminal velocity (three inflorescences each from three plants). We followed the protocol for Sullivan et al. (2018) to measure terminal velocity by dropping seeds down a measurement tube containing two arrays of LED lights and sensors to estimate the speed of seed falls. We conducted seven trial drops of pooled sagebrush seeds using either 500 or 1000 seeds per drop. Terminal velocity measurements ranged from 0.19 to 2.11 m s^−1^. We selected the median terminal velocity of 0.41 m s^−1^ for use in our models.

### Data analysis

Our modelling approach was composed to two parts. The first part involved fitting simplified negative binomial regressions to determine which sources of landscape variance best explained trapped seed density. The second part involved combining an empirical bivariate Student’s t (2Dt) dispersal model (Clark et al. 1999) with the mechanistic Wald analytical long-distance dispersal (WALD) model (Katul et al. 2005) to estimate a latent variable for ground distance traveled of seeds caught above the ground (described below). Fitting models to quantify the influence of scale in a generalized linear model framework (negative binomial regression) enabled us to leverage a well-understood statistical approach to test covariate importance and develop random effect structures (Warneke et al. 2022) for our field data.

### How far do sagebrush seeds disperse and how variable is sagebrush seed dispersal?

We calculated the seed density (‘seeddens’) for each trap by dividing the number of seeds caught by trap area and a standardized term for the number of days deployed (stdays). The standardized day term (stdays) was calculated for each trap as the number of days deployed over the maximum number of days any trap was deployed (*n* = 49). After calculating the seed density for each trap, we calculated the relative standard error (RSE) of seed density for each trap distance across sites, years and patches. Relative standard error is calculated as the standard error over the mean seed density for each distance. Typically, effects with an RSE > 20 % are considered highly variable in ecology (McCune and Grace 2002).

### Which landscape scales best explain variation in trap seed density?

We fit negative binomial regressions using the R package *brms* (Bürkner 2017) of trap seed density as a function of capture height, capture distance and total available seed. The overall model is described as follows:


$$\begin{array}{l} Nseeds\;∼\;negbin(\mu ,\;\phi ) \end{array}$$ (1)

where the number of seeds (Nseeds) is a random variable drawn from a negative binomial distribution, with mean *μ* and overdispersion parameter *∅*.


$$\begin{array}{ll} Log(\mu )=\; & \gamma 0+\;\gamma 1*ht+\gamma 2*dist\\ & +\gamma 3*ht*dist+\gamma 4*fecund\\ & +\;log(stdays)+\log(Area) \end{array}$$ (2)

In Equation (2), *γ*0, *γ*1, *γ*3 and *γ*4 are fitted parameters. *ht* is the capture height, *dist* is the capture distance and *fecund* is the total available seed in the patch. An interaction term between *ht* and *dist* is included. The total available seed term is described as:


$$\begin{array}{l} fecund=seeds_{p}*nrem \end{array}$$ (3)

where *seeds*_p_ is the average maximum seed production per plant and *nrem* is the number of reproductive plants per patch. The trap area (*Area*) and *stdays* term function as offsets (Hilbe 2011), constant terms that scale the mean based on sampling effort.

To determine how trapped seed density varied across different landscape scales, we fit different versions of the basic model, allowing *γ*0, *γ*1, *γ*2 and *γ*3 to vary by group levels as follows:

(1) No landscape effects
(2) Site only
(3) Site × Patch
(4) Patch only
(5) Patch × Transect
(6) Transect only
(7) Site × Patch × Transect

No site was monitored across both years (the location of the trapping at Soda in Year 2 was in a completely separate part of the fire), so ‘Site’ actually refers to a site–year effect. Total available seed, distance and trapped height were all cantered around 0 and scaled by 1 SD to improve convergence. We calculated the leave-one-out cross-validation metric using the *loo* package to compare models with different variations in slope. Model convergence was assessed by assuring all $\hat{r}$ values were no greater than 1.05 and visual inspection of chain mixing (Monnahan et al. 2017). Priors are given in Table B1.

### Do wind-direction metrics help explain variation in seed dispersal?

We considered if wind direction could help explain variation in seed dispersal. We reviewed wind data from the closest NOAA weather station to each site and determined the dominant wind directions of gusts greater than or equal to 32 km h^−1^ during the trapping time (Table 2). Assuming that traps set at angles 180° from the dominant wind direction (i.e. facing the wind) would be most likely to collect seeds, we recorded the smallest absolute difference between the transect angle and the direction the dominant wind gusts were blowing towards. The wind orientation was then scaled (for each value, we subtracted the mean and multiplied the SD) and given as the variable *windorient*. This wind effect was described by a new parameter, *γ*5, which we added as an additional effect to the best-fitting landscape model. The updated Equation (2) for the model with wind effect is then:

**Table 2.** Dominant wind direction for gusts > 32 km h^−1^ (given in degrees) during the trapping dates at the NOAA weather station closest to the site. Alkie was excluded due to seed crop failure and no seeds trapped.

Site and year	Dominant wind direction (°) for gusts > 32 km h^−1^	NOAA weather station
Soda Year 1	200, 250–270	Rome
Botanical Garden Year 1	120–140, 160–170	Boise Airport
Soda Year 2	220–240, 160–170	Rome
Table Rock Year 2	120–140	Boise Airport
Pony Year 2	100–130, 290–310	Mountain Home


$$\begin{array}{ll} \log(\mu )=\; & \gamma 0+\;\gamma 1*ht+\gamma 2*dist\\ & +\gamma 3*ht*dist+\gamma 4*fecund+\;\gamma 5*windorient\\ & +\;log(stdays)+\log(Area) \end{array}$$ (4)

We also considered wind direction as a binary variable with traps either facing towards (within 45° facing a dominant wind direction) or away from the wind as variable windbinary. In this model, the wind effect (*windface*) was described by the parameter, *γ*6.


$$\begin{array}{ll} \log(\mu )=\; & \gamma 0+\;\gamma 1*ht+\gamma 2*dist+\gamma 3*ht*dist\\ & +\gamma 4*fecund+\;\gamma 6*windface\\ & +\;log(stdays)+\log(Area) \end{array}$$ (5)

### How does seed dispersal from remnant patches compare with aerial seeding rates?

We combined a 2Dt empirical dispersal kernel (Clark et al. 1999) with a mechanistic WALD dispersal kernel (Katul et al. 2005). The 2Dt kernel is a bivariate model used to describe decreasing seed or recruit density as distance from the seed source increases and fits using empirical data, while the WALD kernel is a mechanistic model describing the expected movement of a seed in the wind given an understanding of wind movement and seed properties. Our resulting fusion model was used to simulate landscape-scale dispersal of sagebrush seeds. The 2Dt kernel was chosen over other dispersal kernels through an initial exploration looking at capture distance (Bayesian Information Criteria and Akaike Information Criteria kernel comparisons are given in Appendix 1). Similar to other studies of long-distance dispersal, the maximum distance of seed traps was limited by logistical constraints. Fusing lab-based estimates of wind dispersal via the WALD model with our 2Dt dispersal model, informed by field data, enabled us to develop dispersal predictions that made full use of our vertical trap design. In this study, the WALD parameters were set (i.e. we did not propagate uncertainty in wind speed, canopy density or terminal velocity).

The overall model is described as follows:


$$\begin{array}{l} Nseeds\;∼\;negbin(\mu ,\;\phi 2) \end{array}$$ (6)

$$\begin{array}{l} \mu =Area*disp*\left(\;f*\frac{fecund}{1000}\right)*stdays \end{array}$$ (7)

$$\begin{array}{l} disp=a_{transect}\left(\frac{1}{\pi b_{transect}*{\left(1+\frac{dist_{ground}^{2}}{b_{transect}}\right)}^{a_{transect}+1}}\right) \end{array}$$ (8)

*a*_transect_ and *b*_transect_ are fitted parameters that determine the shape of the 2Dt kernel allowed to vary by transect where;


$$\begin{array}{l} a_{transect}=a+\;\omega_{transect}*\nu 1 \end{array}$$ (9)

$$\begin{array}{l} b_{transect}=b+\;\delta_{transect}*\;\nu 2 \end{array}$$ (10)

*a* and *b* are the global parameters for the 2Dt kernel, *ω*_transect_ and *δ*_transect_ are the deviation of each transect from *a* and *b*, respectively, and *ν*1 and *ν*2 are the transect-level variance for the *a* and *b* parameters.

*f* is a fitted parameter describing the effect of total available seed on seed density. Total available seed was divided by 1000 to scale it for model convergence. *Dist*_ground_ is the estimated latent ground distance of a seed caught at a certain height on a trap (i.e. the distance we expected a seed to travel to the ground based on its captured height at a certain distance). *Dist*_ground_ was set at the trap distance (*dist*_trap_) for seeds caught below 20 cm in height (we assumed the additional distance these seeds would travel would be negligible). For seeds caught 20 cm above the ground or higher:


$$\begin{array}{l} dist_{ground}=dist_{trap}+dist_{wald} \end{array}$$ (11)

$$\begin{array}{l} dist_{wald}\;∼\;Wald(\rho ,\;\lambda ) \end{array}$$ (12)

where *ρ* and *λ* are parameters calculated from wind speed, vegetation canopy height, canopy density and terminal velocity. Katul et al. (2005) and Sullivan et al. (2018) describe the calculation of these parameters, including validation with post-dispersal data on spatial patterns of seedling recruitment.


$$\begin{array}{l} \rho =\;{\left(\frac{ht}{\sigma }\right)}^{2} \end{array}$$ (13)

where *ht* is the height of seed capture. *σ* is a parameter calculated as:


$$\begin{array}{l} \sigma^{2}=kc\left(2\frac{\sigma_{w}}{U}\right) \end{array}$$ (14)

where *k* is a scaling coefficient set between 0.3 and 0.4 to describe canopy density. We set *k* at 0.38 for sparse, heterogeneous canopies typical of post-fire systems. *c* is the canopy height surrounding the patch based on our visual estimates from the field at each specific patch. We set *σ*_*w*_ (a measure of boundary conditions) to half of *U* based on Sullivan et al. (2018). *U* is the average daily maximum wind speed during the time periods in which the traps were deployed taken from the closest NOAA or RAWS weather station.


$$\begin{array}{l} \lambda =\;\frac{htU}{V} \end{array}$$ (15)

where *V* is the terminal velocity of sagebrush seeds. Priors are given in Table B2.

After fitting the combined empirical mechanistic model, we created a forward version in R that sampled from the posterior distributions of our parameters and ran 10 000 simulations to estimate seed dispersal at distances between 0 and 100 m.

## Results

### How far do sagebrush seeds disperse, and how variable is sagebrush seed dispersal?

**Figure 2.**
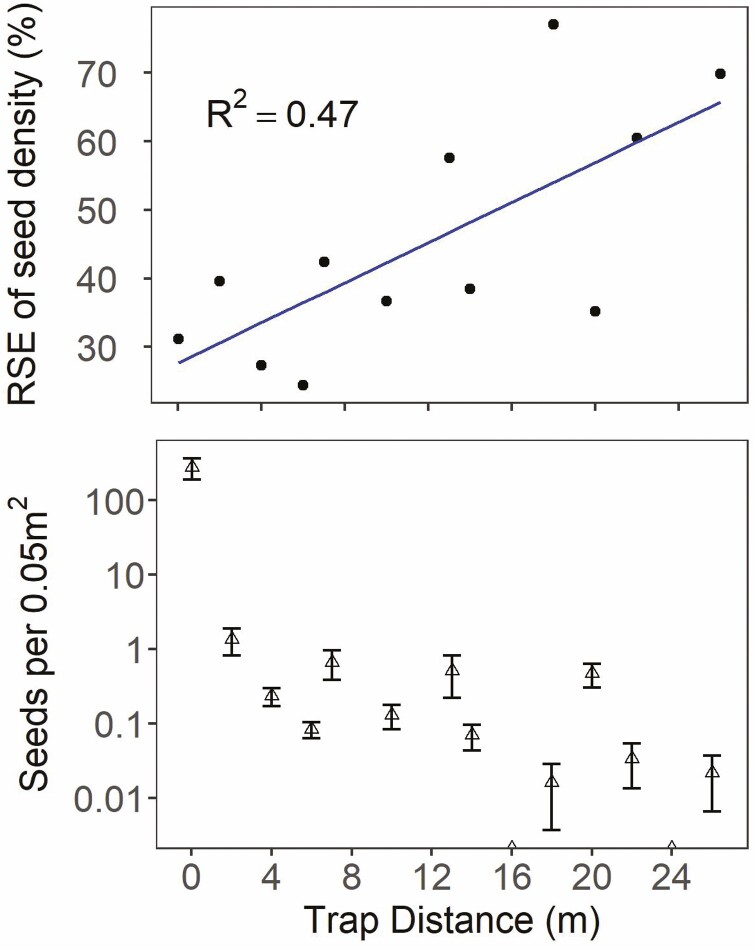
Relationship of mean trap abundance (bottom panel) and variability (RSE, top panel) of the density of seeds captured (per 0.05 m^2^ of vertical trap area) relative to the distance of seed traps from seed source patch. Seed density is standardized by the number of days in each collection interval period shown as the mean per trap ± the standard error (bottom). Alkie was excluded due to seed crop failure and no seeds trapped.

No seeds were caught on vertical seed traps at the Alkie fire, despite extending the trapping time several weeks past the initial ~3-week observation period. Seeds on patch plants appeared not to develop at the Alkie site, and thus it was excluded from analysis. At the other sites, 31 % of traps captured seeds. Two seeds were detected on each on two of the traps at the maximum distance of 26 m from the seed-source patches. Relative standard errors of seed density for each trap distance across sites and years were large, ranging from 24 to 77 % (Fig. 2). Relative standard error tended to increase with distance of traps from seed-source patches (*R*^2^ = 0.47), indicating that dispersal became more variable the farther the distance from the patch.

### Which landscape scales best explain variation in seed dispersal (trap, transect, patch, site)? Do wind-direction metrics help explain variation in seed dispersal?

**Figure 3.**
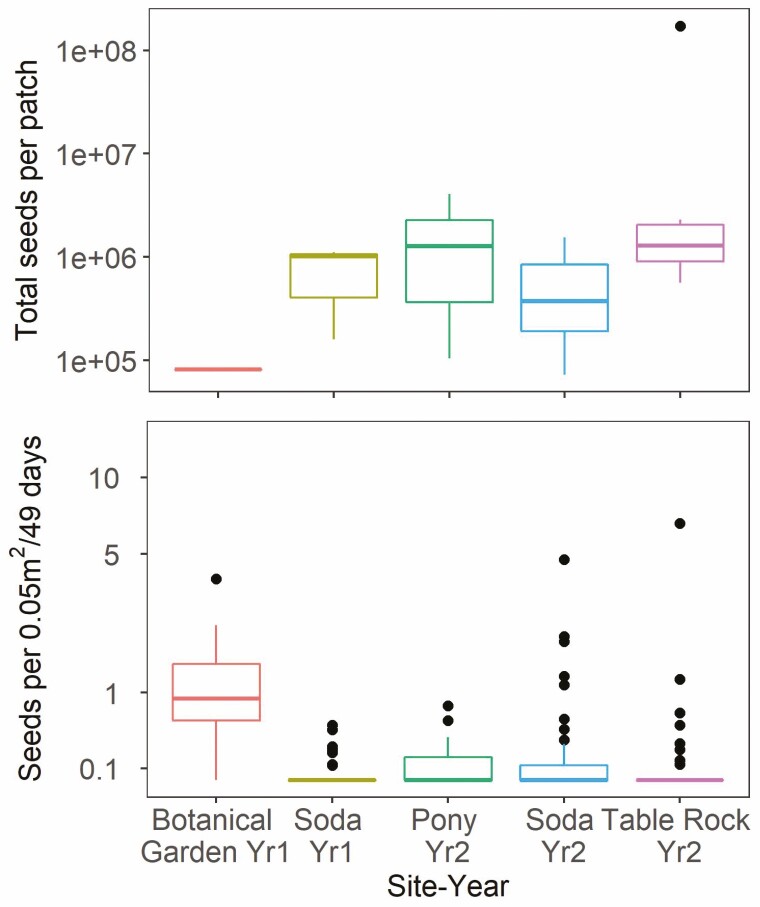
Box plots of the estimated total available seeds per patch (fecundity × number of reproductive plants) across sites (top) and number of seeds across traps of all distances caught per 0.05 m^2^ trap area standardized by 49 days deployed (bottom). The graphs do not include under-crown traps. The unit of measure for the top graph is a patch (*n* = 19) and the unit of measure for the bottom graph is a trap (seed counts aggregated across heights, *n* = 273). Alkie was excluded due to seed crop failure and no seeds trapped.

**Figure 4.**
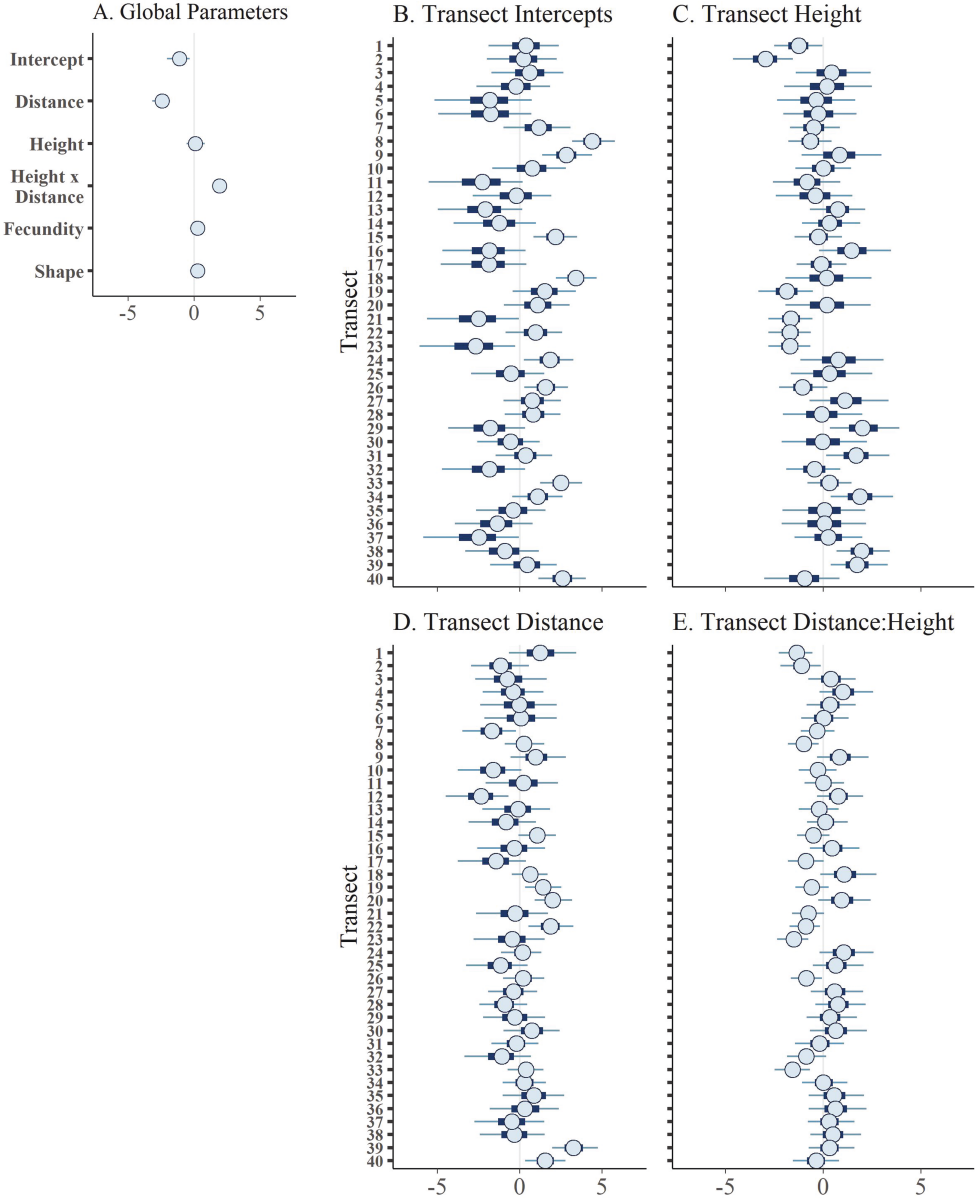
Posterior distributions intervals for parameters of the landscape negative binomial seed density model with intercepts and slopes varied by transect. The centre circle of each distribution shows the median, the thick bars show the 50 % credible interval and the thin lines show the 90 % credible interval. Predictors were scaled prior to analysis so that parameter values represent relative effect size of each predictor on trapped seed density. (A) Global parameters, (B) varying intercepts by transect, (C) varying slope of the height parameter by transect, (D) varying slope of the distance parameter by transect and (E) varying slope of the height:distance parameter by transect.

The total number of available seeds produced by the sagebrush present in each patch varied across years and sites, with the greatest mean total observed at the Table Rock Fire in Year 2 (Fig. 3). However, available seed abundances did not relate to the number of seeds caught per trap, nor were there consistent relationships of available seeds to abundance of seeds captured by seed traps in each patch (90 % credible interval for total available seed [−0.14, 0.66]) (Fig. 4). On average, the most seeds per trap were caught at the lowest elevation site, the Botanical Garden in Year 1.

Model performance increased as the landscape scale of variance decreased with best model performance at the transect level, the finest spatial scale in this study (Table 3). Models incorporating multiple levels of variance did not perform better than models with single lower levels of variance. This indicates that the primary source of spatial variability in seed rain occurred at a small-scale level (different sides of patches) rather than either at the scale of (i) the five sites across 2 years or (ii) patch level.

**Table 3.** Comparison of leave-one-out information criteria between different landscape models.

Model	loo IC
Model 1: No landscape variation	1927.10
Model 2: Site only	1803.70
Model 3: Site × Patch	1795.50
Model 4: Patch only	1780.10
Model 5: Patch × Transect	1770.00
Model 6: Transect	1756.1
Model 7: Site × Patch × Transect	1766.30
Wind Model 1: Wind Angle with Transect	1760.7
Wind Model 2: Binary Wind with Transect	1766.7

**Figure 5.**
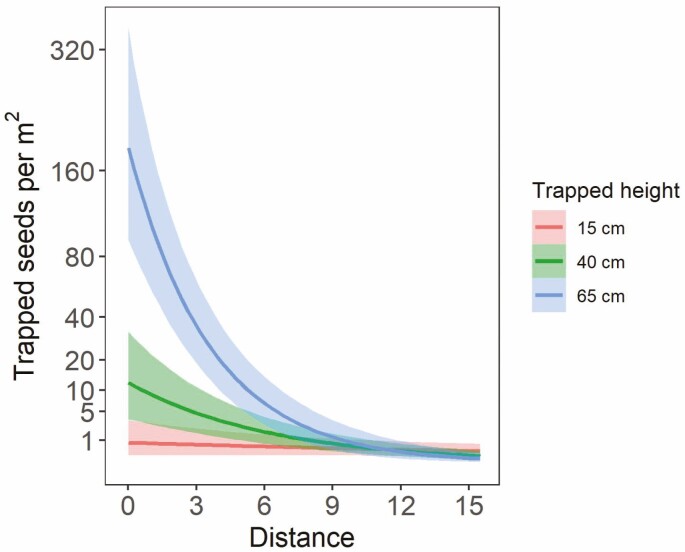
Mean number of trapped seeds per m^2^ area predicted from the landscape model with slope varied by transect, showing the interacting effects of trapped height and trapped distance on seed density. The shaded ribbons show the 90 % credible intervals.

As expected, distance had the strongest effect on trapped seed density (Fig. 4). Estimated seed density decreased from a mean of ~13 seeds per m^2^ [90 % credible interval: 0–66] to <1 seeds per m^2^ [90 % credible interval: 0–4] as distance increased from 0 to 10 m from the source, holding all other predictors constant. Neither total available seed nor height had a consistent effect on trapped seed density (90 % credible intervals crossed zero) (Fig. 4). However, there was a positive interaction between trap distance and trapped height on seed density, with more seeds caught at higher heights at distances near the source (Fig. 5). For example, at a distance of ~0.3 m, more than 160 seeds per m^2^ were predicted to be trapped at 65 cm height, as opposed to 11 or <1 seeds per m^2^ at 40 and 15 cm height, respectively.

The random intercept varied more between transects than did the slope of distance, height or the interaction between distance and height (Fig. 4). This indicates that the effects of distance and height on seed density were less variable between transects than overall seed density differences. Including either continuous or a binary metric of wind direction did not improve model performance (Table 3).

### How does seed dispersal from remnant patches compare with aerial seeding rates?

**Figure 6.**
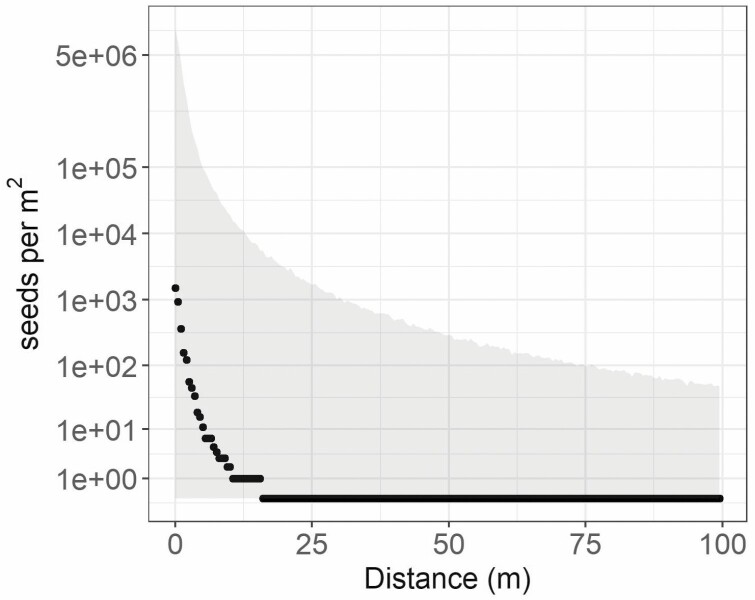
Simulated median seed dispersal (seeds per m^2^) estimated using the seed dispersal model with transect-level variation in dispersal kernel (1000 simulations), based on global parameters for the *p* and *u* parameters of the 2Dt kernel, and assuming an average of 30 000 seeds per reproductive plant, and 25 individuals per patch. The grey ribbon shows the 90 % quantiles of the simulations.

Seed dispersal predicted for a median transect with a fecundity of 30 000 seeds per individual and a patch size of 25 individuals (750 000 total available seeds) would decrease to 0 seeds per m^2^ capture area at a distance of ~16 m distance from the patch, based on the median of 10 000 simulations (Fig. 6). However, in the top 5 % of simulations, there were still 48 seeds per m^2^ at 100 m distance and in the lower 5 % of simulations; there was no dispersal at any distance. These seed dispersal simulations were highly variable. For example, the 90 % quantiles for modelled seed dispersal to 5 m from patches ranged from 0 seeds per m^2^ to >100 000 seeds, and the median value was 12 seeds per m^2^. For comparison, on the Soda wildfire, the aerial sagebrush seeding rate was between approximately 95 and 250 aerial pure live seeds per m^2^.

## Discussion

Seed availability is an important component of restoration and rehabilitation of disturbed areas, particularly for foundational species like sagebrush that can only re-establish from short-lived seeds. Insufficient seeding could cause missed recovery opportunities, while unnecessary seeding of areas with adequate natural seed could waste resources and carry unnecessary collateral ecological risks (e.g. potential introduction of maladapted genotypes, Seaborn et al. 2021). Therefore, there is a pressing ecological need to develop better methods of predicting natural seed dispersal across disturbed landscapes. Our study presents a rare attempt to quantify seed dispersal at management-relevant scales by integrating both empirical and mechanistic modelling. Although our seed dispersal predictions indicated a high degree of uncertainty, they revealed that seed dispersal from unburned remnant sagebrush or actively created sagebrush patches is a major source of variability in natural post-fire regeneration of sagebrush. Even areas very close to these patches may experience limited seed dispersal.

### Landscape variability

Although we found a measurable amount of seed dispersal from sagebrush patches, there was a high degree of variability in dispersal between transects, even when total available seed and patch size were accounted for. Differences in canopy heights and plant densities can strongly affect wind movement and wind-transported seeds (Bohrer et al. 2008; Nuttle and Haefner 2017). These previous studies from forested studies show that strong bursts of vertical wind (influenced by the structure of the canopy) are particularly important to long-distance seed dispersal. In comparison to forests, recently burned sagebrush-steppe ecosystems have minimal canopy structure, and wind movement near the ground is less likely to be strongly affected by remaining vegetation (Driese and Reiners 1997). Furthermore, although we found clear evidence that sagebrush seeds are dispersed by wind, they lack a true wind dispersal mechanism (such as a pappus; in spite of being in the Asteraceae family) that would allow them to remain aloft in vertical wind lifts for extended transport. The predictive strength of models that account for variability at different directions from the patch has implications for theoretical and applied research on seed dispersal, where isotropy (equal probability of dispersal in all directions) is often assumed (van Putten et al. 2012).

Sagebrush steppe often occurs in topographically complex areas, and even though our sampling areas were relatively flat, airflow patterns caused by the surrounding hills could have contributed to the high variability in seed dispersal we observed across different transects. The greater variation in seed dispersal at the transect level than at the site or patch level, combined with the lack of explanatory power of coarse (‘average’) wind-direction metrics suggests that transect identity may have been a proxy for canopy structure, topography and stronger and unaccounted-for wind variability within sites. Due to the difficulty in controlling for these factors in the field, the question of how topography and vegetative structure influences seed dispersal could be addressed in follow-on investigation using mechanistic modelling (Nathan et al. 2009).

Height of seed release is another factor that can contribute to differences in dispersal distances (Thomson et al. 2011; Schupp et al. 2019). In canopies with variable heights of plant crowns (as was the case in our patches), assessing maternal plant height effects on dispersal can be difficult because plants may not contribute equally to seed dispersal, and tracing seeds to specific source plants requires genetic analysis via DNA microsatellites (Ashley 2010). However, the effect of sagebrush height on dispersal distance could be addressed in an experimental context by trapping around individual plants of different heights. In many semi-arid landscapes, mound-like features are created by mammals, insects or geomorphic processes, such as the very common ‘mima mounds’ of sagebrush steppe that host relatively tall and fecund plants elevated above the surrounding sagebrush population (Hill et al. 2005). These microtopographic effects would be important considerations in modelling height of seed release.

Phenology is another important factor in determining seed dispersal by wind. Some tree species with specific wind dispersal mechanisms synchronize seed ripening and release with meteorological conditions that promote long-distance seed dispersal (Heydel et al. 2015). Although species in open vegetative habitats, including many Asteraceae species, do not display such targeted release patterns (Tackenberg et al. 2015), the timing of seed ripening and release can still have an impact on dispersal distances. In our second year of trapping, initial seed development was delayed, possibly due to above-average rain in October. A significant wind event occurred at Table Rock in mid-November during our first 3 weeks of trapping yet there were few seeds collected in traps. Seeds did not appear fully developed or easy to remove from the inflorescences at that time, and appreciable seed capture was not detected until later in December. An improved understanding of how seed development coincides with major wind events may help elucidate differences in patch and site seed dispersal.

### Estimating landscape-scale dispersal distance

Predicting seed dispersal becomes more difficult as distance from the maternal plant increases (Bullock and Clarke 2000; Fig. 2) but can be particularly critical to vegetative recovery in disturbed systems when seed sources are limited (Hammill et al. 1998; Borchert et al. 2003; Urza and Sibold 2017). We attempted to address this problem by utilizing vertical traps, measuring height of seed capture and integrating a mechanistic wind dispersal model into our empirical dispersal kernel to simulate latent ground distance a seed would travel. Our approach allowed us to estimate a range of dispersal distances without actually placing traps at locations where seed dispersal was expected to be so rare that we were unlikely to detect it. We believe this approach could be further refined and used to estimate landscape-scale wind dispersal of other species of restoration or conservation concern. The key point is that height of seed capture can be used as a proxy by which to estimate dispersal distance, if certain properties of the seed and system are known (seed terminal velocity, average wind velocity, canopy density). We used a modestly parameterized approximation of a WALD dispersal kernel in this study and incorporating microsite-specific wind measurements and site-specific terminal velocity metrics could further improve predictions (Sullivan et al. 2018).

On the Soda wildfire, widespread aerial sagebrush seeding of a rate between ~95 and 250 aerial pure live seeds per m^2^ (not applied at the time of our study) generally overcame seed limitations to allow for significant seedling establishment in the first year after fire (Germino et al. 2018). Establishment was strongly limited by topographic features, absence of ‘fertile islands’ (high organic-content areas where sagebrush existed pre-fire and burned) and dominance of exotic annual or perennial grasses (Germino et al. 2018). While our seed dispersal models show that it is possible that remnant sagebrush islands could generate as much seed as aerial seeding in some rare instances close to the patch, it is highly unlikely that this seed dispersal would reach the microsites needed for significant population re-establishment.

One further consideration is the potential role of negative density dependence inside and near remnant sagebrush patches (Zaiats et al. 2020). Given that the majority of sagebrush seeds fall within a few metres of the mother plant, many of the seeds will be establishing with the zone of influence of not only the mother plant but possibly other individuals in the patch. Strong negative density dependence is likely to further negate the seed contribution of remnant sagebrush patches to landscape-scale sagebrush regeneration.

## Conclusions

Developing quantitative models for spatial prioritization of restoration efforts is a major research objective with immediate applicability to land management. Small-scale and near-term forecasting of vegetative regeneration is an integral part of making decisions about where and when to actively manage landscapes (Dietze et al. 2018). In this study, we demonstrated how empirical and mechanistic dispersal models can be integrated to predict post-fire seed dispersal from undisturbed seed sources and that large burned areas in sagebrush steppe likely receive little or no natural sagebrush seed deposition across most of their area. These results can be utilized in predictions of post-fire regeneration for determining which areas of the landscape to actively manage.

## Supporting Information

The following additional information is available in the online version of this article—

**Figure S1.** A photo of seed traps set up along transects at the Soda wildfire.

plac045_suppl_Supplementary_Figure_S1

## Data Availability

Data will be released with publication of this paper to the Forest Service Research Data Archive and can be accessed here: https://www.fs.usda.gov/rds/archive/Catalog/RDS-2021-0073. Code for the models is available here: https://zenodo.org/badge/latestdoi/537824506.

## Notes

**Populations & Communities. Chief Editor:** Jean Burns

## Appendix 1

**Table A1.** Comparisons of different dispersal kernel fits using distance-only (no height) fit using maximum likelihood.

Fit	Source	AIC	BIC
Gaussian	Clark et al. (1999)	1334.45	1345.75
Ribbens	Ribbens et al. (1994)	1355.87	1367.18
Negative exponential	Greene and Calogeropoulos (2002), Bullock and Clarke (2000)	1279.39	1290.7
2Dt	Clark et al. (1999)	1201.55	1216.62
Inverse power	Bullock and Clarke (2000)	1205.63	1220.703

## Appendix 2

**Table B1.** Priors on parameter values for the landscape model.)

Parameter	Description	Prior
*γ*0	Intercept	student_t(3, −2, 10)
*γ*1	Effect of height on trapped seed density	normal(0, 1)
*γ*2	Effect of distance on trapped seed density	normal(0, 1)
*γ*3	Height × distance interaction effect on seed density	normal(0, 1)
*γ*4	Effect of total available seed on trapped seed density	normal(0, 1)
*∅*1	Dispersion parameter for negative exponential	exponential(1)
*sd_transect*	A set of variance parameters describing transect-level variance height and distance parameters (*γ*1, *γ*2, *γ*3)	student_t(3, 0, 10

**Table B2.** Priors on parameter values for the empirical 2Dt and mechanistic WALD integrated model. Priors on *ω*, *δ*, *ν*1 and *ν*2 were set weighted more strongly towards 0 with the assumption that transects would not display extremely different dispersal kernels.

Parameter	Description	Prior	Constraint
*a*	Global parameter governing 2Dt kernel	half-normal(0, 1)	*a* ≥ 0
*b*	Global parameter governing 2Dt kernel	half-normal(0, 1)	*b* ≥ 0
*f*	Total available seed effect	half-normal(0, 1)	None
*∅*2	Dispersion parameter for negative exponential	exponential(1)	*∅* > 0
*ω*	Deviation between *a* and each transect	normal(0, 0.5)	None
*δ*	Deviation between *b* and each transect	normal(0, 0.5)	None
*ν*1	Transect-level variance for the *a* parameter	exponential(4)	*ν*1 > 0
*ν*2	Transect-level variance for the *b* parameter	exponential(4)	*ν*2 > 0
*dist*_wald_	Latent distance travelled between the trap and the ground	wald(*ρ*, *λ*)	0 ≥ *dist*_wald_ ≥ 50
