# Supplementary figures and images for: Post-fire seed dispersal of a wind-dispersed shrub declined with distance to seed source, yet had high levels of unexplained variation
Source: AoB Plants. 2022 Oct 6;14(6):plac045. doi: 10.1093/aobpla/plac045 (PMC9661893; doi:10.1093/aobpla/plac045)

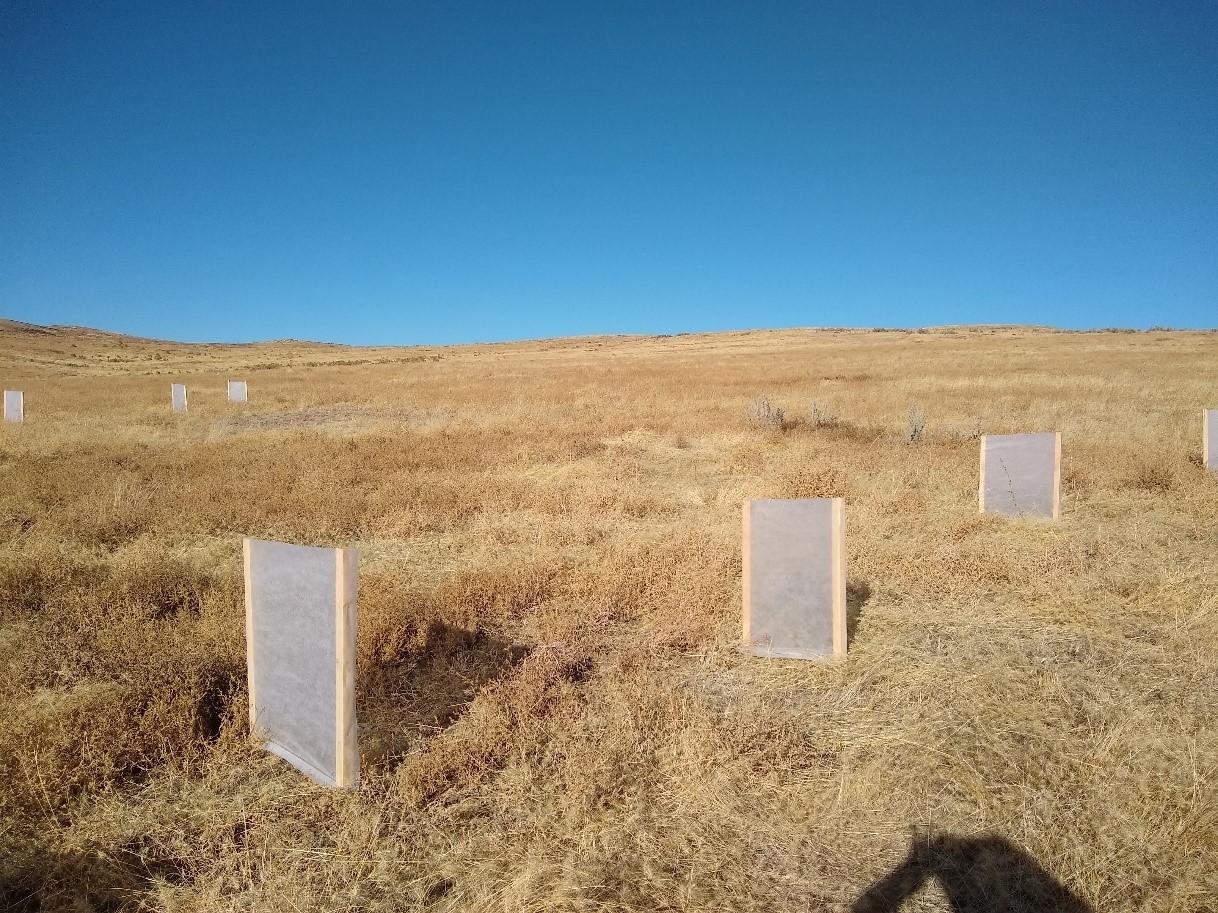

Supplement: plac045_suppl_Supplementary_Figure_S1 [file plac045_suppl_supplementary_figure_s1.jpeg]
